# Supplementary material for: WordCommentsAnalyzer: A windows software tool for qualitative research
Source: F1000Res. 2018 Sep 4;7:536. Originally published 2018 May 3. [Version 2] doi: 10.12688/f1000research.14819.2 (PMC6137411; doi:10.12688/f1000research.14819.2)
Supplement: Supplementary file 3 [file f1000research-7-17614-s0002.tgz › b2a79496-4ada-47c4-9682-a99ce867836d.zip!/Eight Iranian Health Journals/Salmand_Iranian Journal of Ageing _30.docx]

© Salmand_Iranian Journal of Ageing . This work is licensed under CC BY-NC 4.0 (https://creativecommons.org/licenses/by-nc/4.0/).

Volume & Issue: دوره 6 -

Title: الگوی تجویز دارو در سالمندان مقیم آسایشگاه های شهر تهران (مقاله برگرفته از پایان نامه دانشجویی)

http://salmandj.uswr.ac.ir/article-1-478-fa.html

اهداف: افراد سالمند در قیاس با دیگر گروه های سنی،بیشتر به بیماری مبتلا شده و دارو مصرف می کنند.با توجه به آسیب‌پذیری سالمندان مقیم آسایشگاه، مصرف دارو در آن‌ها اهمیت بیشتری پیدا می‌کند. این مطالعه با هدف بررسی تعیین الگوی تجویز دارو در سالمندان مقیم آسایشگاه‌های شهر تهران انجام شده است. مواد و روش‌ها: در این مطالعه مقطعی، اطلاعات دارویی 170 نفر سالمند مقیم آسایشگاه، که به روش نمونه گیری خوشه‌ای تصادفی دو مرحله‌ای انتخاب شده بودند، استفاده شد. برای جمع‌آوری داده‌ها از پرونده بیماران و مصاحبه با پرستار وپزشک و تکمیل پرسشنامه مورد نظر استفاده گردید. داده‌های حاصل با استفاده از شاخص‌های میانگین و پراکندگی توصیف، و با استفاده از آزمون‌های مجذور خی، تی تست، و فیشر تحلیل شد. برای این منظور نرم‌افزار آماری SPSS نسخه 16 به‌کار گرفته شد. یافته‌ها: میانگین سنی سالمندان نمونه گیری شده 2/4±79/75 سال، و 64/7درصد آنها مونث بودند. 62/4درصد بیش از 5 قلم دارو دریافت می‌کردند. میانگین اقلام دارویی تجویز شده 7/55 بوده و دامنه اقلام از 1 تا 19 گسترده بود. قرص‌ها با 98/2درصد، داروهای تزریقی با 20/6درصد، قطره‌ها با 13/5درصد، شربت با 8/8درصد، اسپری با 6/5درصد و پماد و شیاف با 2/9درصد بیشترین اشکال دارویی مصرف شده بودند. گروه دارویی سیستم اعصاب مرکزی بالاترین درصد فراوانی مصرف را داشت و پس از آن ویتامین‌ها، داروهای قلبی-عروقی، داروهای خونی(ضد انعقادها) و داروهای گوارشی قرارداشتند. پرمصرف‌ترین داروها عبارت بودند از ویتامین‌ها (81/2درصد)، آسپرین (37/64درصد) و آلپرازولام (22/35درصد). بین گذراندن دوره‌های آموزشی سلامت سالمندان و میانگین اقلام دارویی تجویز شده رابطه معنی‌داری وجود نداشت (P=0/054). دارا بودن تخصص پزشکی در میانگین اقلام دارویی تجویز شده تفاوتی را ایجاد نمی‌کرد (P=0/068)  ولی ارتباط معنی‌داری بین داشتن و نداشتن بیمه و تعداد اقلام دارویی تجویز شده وجود داشت (P=0/05). نتیجه‌گیری: تدوین و برگزاری برنامه‌های آموزشی داروشناسی سالمندی برای پزشکان ونظارت بیشتر بر روی مراکز مراقبت از سالمندان می تواند تاثیر مناسبی در اصلاح فرهنگ تجویز دارو داشته باشد.

Title: الگوی تجویز داروهای سرپایی در سالمندان تحت پوشش سازمان تأمین اجتماعی در سال 1388 (مقاله برگرفته از پایان نامه)

http://salmandj.uswr.ac.ir/article-1-475-fa.html

اهداف: افزایش امید به زندگی و رشد روز افزون سالمندان بعنوان تهدید و فرصت توجه محققین را به خود جلب نموده است. مطالعات حکایت از افزایش هزینه‌های درمانی و مراقبت از سالمندان نسبت به سایر گروه‌های سنی دارد. هدف این مطالعه بررسی الگوی تجویز دارو در سالمندان تحت پوشش سازمان تامین اجتماعی ایران و مقایسه آن در گروه‌های سالمندی بوده است. مواد و روش‌ها: این مطالعه از نوع توصیفی، تحلیلی و مقطعی بوده که به‌صورت گذشته نگر با استفاده از نسخ دارویی مراجعین سرپایی تحت پوشش سازمان تامین اجتماعی به داروخانه‌های طرف قرارداد در طی سال 1388 و ثبت شده در دفاتر رسیدگی به اسناد پزشکی سازمان تامین اجتماعی انجام شده است. اطلاعات مشتمل بر جداول پایه تعداد دارو، الگوی میزان تجویز دارو، الگوی میانگین ریالی و هزینه تجویز دارو، الگوی گروه های دارویی پرمصرف بر اساس محل آناتومیک اثر دارو (Anatomic Therapeutics Chemical)، الگوی گروه‌های دارویی پرمصرف به تفکیک گروه سنی سالمندان و گروه غیرسالمند و به تفکیک گروه‌های سنی سالمندی بر اساس تعریف سازمان بهداشت جهانی توسط محقق بر اساس میزان دسترسی تعریف شده توسط سازمان استخراج شده است. یافته‌ها: الگوی میانگین تجویز دارو در دو گروه زیر 60 سال و بالای 60 سال اختلاف معنی‌دار را نشان می‌دهند.(P<0/005) الگوی میانگین تجویز دارو در سه گروه سالمندی اختلاف معنی‌دار دارد. (P<0/001) الگوی میانگین هزینه تجویز دارو در دو گروه زیر 60 سال و بالای 60 سال اختلاف معنی‌دار را نشان می‌دهند. (P<0/004) الگوی میانگین هزینه تجویز دارو در سه گروه سالمندی متفاوت است واین اختلاف معنی‌دار است. (P<0/001) نتیجه‌گیری: بر اساس نتایج به‌دست آمده از این مطالعه، سالمندی بر میزان بار مراجعه به پزشک و داروخانه اثر مستقیم و تزایدی دارد و در بین گروه‌های سالمندی با افزایش سن میزان هزینه‌ها سیر صعودی داشته ولی میزان بار مراجعه کاهش می‌یابد. که می‌توان پس از شناسایی بیشترین داروهای مصرفی سالمندان با آموزش خانواده‌ها از ابتلاء به بیماری‌های مزمن پیشگیری نمود و با آموزش سالمندان و پیشگیری از عوارض دارویی زندگی کم عارضه‌تری را برای سالمندان مهیا نمود.

Title: بررسی ارتباط عوامل اجتماعی و جمعیت‌شناختی با بیماری‌های شایع در کارکنان سالمند صدا و سیمای مرکز تهران در سال 1389 (مقاله برگرفته از پایان نامه)

http://salmandj.uswr.ac.ir/article-1-476-fa.html

اهداف: پژوهش حاضر با هدف بررسی ارتباط اجتماعی و جمعیت‌شناختی با بیماری‌های شایع در کارکنان سالمند صدا و سیمای مرکز تهران انجام شد. با توجه به روند افزایش سالمندی در جهان و ایران، شیوع ناتوانی های جسمی در سالمندان افزایش یافته که پیامدهایی مانند بستری شدن، استفاده روز افزون از سیستم‌های مراقبتی–بهداشتی و مرگ‌و‌میر را به‌دنبال دارد. در راستای دستیابی به سلامت سالمندان و پیشگیری از بروز و ابتلای آن‌ها به بیماری‌های مزمن و کاهش شیوع بیماری‌های ویژه در آن‌ها، نخستین گام، شناسایی وضعیت سلامت آنان است. تا بدین ترتیب بتوان نیازهای بهداشتی و درمانی آنان را مشخص نمود و با شناسائی بیماری‌های همراه سالمندی اقدام به پیشگیری (کنترل آن) کرد. هم چنین، در محدوده بررسی‌ها از سوی نگارنده برای یافتن پژوهشی در زمینه ارتباط عوامل دموگرافیک با شیوع بیماری‌ها در مراکز رادیویی و تلویزیونی نتیجه ای به‌دست نیامد. مواد و روش‌ها: مطالعه حاضر یک مطالعه توصیفی مقطعی جمع‌آوری نمونه ها به صورت تمام شماری بوده که بر روی 152 سالمند (بدون اختلالات شناختی) که به کلینیک صدا وسیمای مرکز تهران مراجعه نموده بودند انجام شد. در این پژوهش از پرسشنامه پژوهشگر ساخته که در دو بخش تنظیم شده بود، (یک بخش اطلاعات دمو گرافیک و بخش دیگر سابقه ابتلای بیماری‌ها) استفاده شد. سپس بیماران توسط پزشک ویزیت شده و جهت تکمیل اطلاعات مربوط به سابقه بیماری، از پرونده‌های پزشکی موجود در بایگانی کلینیک استفاده شد. یافته‌های حاصل توسط نرم افزار spss16 با استفاده از روش‌های آماری تی تست و کای دو و ضریب همبستگی پیرسون مورد تجزیه و تحلیل قرار گرفت. یافته‌ها: براساس نتایج تحقیق، شایع‌ترین بیماری‌ها استو آرتریت (46درصد)، پرفشاری خون (36درصد) بیماری‌های قلبی (35/5درصد) و دیابت (21درصد) بودند. در این مطالعه بین بیماری‌های استوآرتریت، پرفشاری خون و بیماری‌های قلبی با سن (P=0.001)، (BMI (P=0.000، میزان تحصیلات (P=0.000)، میزان درآمد (P=0.000) و فعالیت ورزشی (P=0.01) ارتباط آماری معنی‌دار مشاهده گردید. نتیجه‌گیری: بیماری استوآرتریت، بیماری‌های قلبی، پرفشاری خون و دیابت از شایع ترین بیماری‌های دوران سالمندی در کارکنان سازمان صدا و سیما می‌باشند. یافته‌ها نشان داد که بین این بیماری‌ها و برخی متغیرهای نشان‌دهنده وضعیت سلامت در این مطالعه مانند سن، میزان تحصیلات، فعالیت ورزشی و BMI ارتباط مثبت وجود دارد. بنابراین با تأکید بر ضرورت تدوین سیاست‌های اصولی برای شناخت ریشه‌ای بیماری‌ها، با اجرای برنامه‌هایی مانند تغییر در شیوه زندگی،‌ انجام تمرینات ورزشی منظم و مداوم، تغییر عادات غذایی و معاینات منظم دوره‌ای می‌توان بیماری‌های فوق را شناسائی نمود و از ناتوانی سالمند جلوگیری به عمل آورد.

Title: بررسی تأثیر سم‌زدایی بر کیفیت زندگی معتادان 65-40 ساله مراجعه‌کننده به انجمن خیریه احیای انسانی

http://salmandj.uswr.ac.ir/article-1-480-fa.html

اهداف: در طی دو دهه اخیر فعالیت‌های مربوط به کاهش تقاضا و به‌ویژه درمان و بازتوانی معتادان در ایران ارتقائ چشمگیری یافته است. هم اکنون آلترناتیوهای متعدد درمانی از جمله مراکز درمان سرپایی و بستری، روش‌های پرهیز مدار و ایمان مدار از جمله معتادان گمنام و مراکز خیریه تولدی دوباره به‌صورت دارویی و غیر دارویی فعالیت می‌کنند. روش سم‌زدایی و درمان در جمعیت خیریه احیای انسانی (کنگره 60) به‌صورت انحصاری بوده و با جایگزینی شربت تریاک و کاهش تدریجی آن (طی 11 ماه) و همچنین گروه درمانی و معنویت درمانی می‌باشد. پژوهش مذکور مطالعه‌ای، توصیفی است که در مدت3 ماه (بدو ورود،یکماه و سه ماه بعد درمان) به‌صورت طولی و هم‌گروهی انجام شده است. در این پژوهش کیفیت زندگی در سه نوبت مورد ارزیابی قرار گرفته است. افراد حاضر در تحقیق شامل تمامی معتادان بالای 40 سالی است که به این انجمن جهت سم زدایی و درمان مراجعه نموده اند. انتخاب نمونه‌ها به روش نمونه‌گیری در دسترس بوده است. ابزار گردآوری اطلاعات پرسشنامه محقق ساخته اطلاعات جمعیت شناختی، پرسش‌نامه کیفیت زندگی SF36 می‌باشد، سپس داده‌ها با نرم افزار SPSSمورد تجزیه و تحلیل قرار گرفته‌اند. یافته‌ها: از مجموع 33 نفر، 15 نفر بالای 50 سال و 3 نفر بیشتر از 60 بوده اند. ماده سوء مصرفی شامل تریاک با فراونی 12 نفر (%36/4)، کراک و هروئین 14 نفر (%42/2)، بالاترین درصد شیوع را داشتند. میانگین ماهیانه هزینه سوء مصرف مواد برای هر نفر به‌طور متوسط 159/849 تومان می باشد. برای تمامی زیر مقیاس‌های کیفیت زندگی،P  زیر 0/05 گزارش گردیده است. نتیجه‌گیری: سم‌زدایی در ارتقای کیفیت زندگی است موثر بوده است و فقط برای دو سئوال "به نظر می‌رسد که من نسبت به دیگر افراد راحت تر مبتلا به بیماری می شوم" و "سلامتی من مثل دیگر افرادی است که می‌شناسم" نظر فرد در سه دوره تغییر نکرده است. شاید بتوان گفت که درمان‌های کوتاه مدت بر روی تغییر "نگرش عمیق فرد نسبت به خود" کم اثر باشد و تغییر نگرش بنیادی و و عمیق نیاز به برنامه‌های بازتوانی طولانی‌مدت‌تری داشته باشد.

Title: تبیین تجربه سالمندان و مراقبین عضو خانواده آنان از دوستدار سالمندبودن شهر تبریز (مقاله برگرفته از پایان نامه دانشجویی)

http://salmandj.uswr.ac.ir/article-1-477-fa.html

اهداف: این تحقیق به منظور بررسی تجارب سالمندان و مراقبین عضو خانواده شهر تبریز در خصوص وضعیت دوستدار سالمند بودن شهر تبریز انجام گرفت تا امکان ارایه پیشنهادات کاربردی برای ارتقای وضعیت شهر مهیا گردد. مواد و روش‌ها: این پژوهش به صورت مطالعه کیفی در سال 1389 در 4 مرکز بهداشتی درمانی ‌کلان‌شهر تبریز انجام یافت. در این مطالعه 4 بحث گروهی جداگانه با حضور سالمندان یا مراقبین عضو خانواده آنان‌ انجام و کلا 32  نفر در این بحث‌ها حضور داشتند. یافته‌ها: درمطالعه حاضر، نظرات در طبقات خانه، حمل و نقل و معابر، پارک‌ها جمع‌آوری گردید. در کل بیشترین تجربه از وجود موش‌های فاضلاب و وضعیت حمل و نقل اتوبوس شهری بود. در مرکز منظریه بیشترین تجربه ناخوشایند از حمل و نقل و ایستگاه‌های اتوبوس اعلام گردید. در مرکز منتظری وجود موش‌های فاضلاب و مشکلات معتادان در پارک عنوان شد. در مرکز جمشید عدم وجود سیستم فاضلاب عمده‌ترین نگرانی بود. در مرکز زعفرانیه مسیر بد اتوبوس و حیوانات موذی مهم‌ترین مشکل ذکر شد. نتیجه‌گیری: استانداردسازی حمل و نقل درون‌شهری، کاهش هزینه‌های تاکسی، تامین سیستم دفع فاضلاب، مبارزه باحیوانات موذی، آموزش‌های‌ رسانه‌ای، نصب تابلوهای ویژه‌ سالمندان، تعبیه صندلی درخیابان‌ و محله‌ها، استانداردسازی پارک‌ها، فراهم‌کردن امنیت‌ ازجمله فعالیت‌هایی است که باید با مشارکت سیاست‌گذاران، مردم و سالمندان در راستای دوستدار سالمند شدن محیط زندگی آنان‌ صورت پذیرد.

Title: تبیین مفاهیم مرتبط با سالمند‌آزاری در ادبیات فارسی (مقاله برگرفته از پایان نامه دانشجویی)

http://salmandj.uswr.ac.ir/article-1-482-fa.html

اهداف: سالمندآزاری (انجام یا عدم انجام یک رفتار منفرد یا تکرار شونده که باعث اذیت یا آسیب‌رساندن به یک سالمند شود) یکی از معضلات سلامت اجتماعی در جوامع امروزی به شمار می‌رود و در پیشگیری از آن آموزش نقش برجسته ای دارد. هدف از پژوهش حاضر بررسی مفاهیم مرتبط با سالمندآزاری در آثار مکتوب ادبیات فارسی بود. مواد و روش‌ها: در این پژوهش مضامین مرتبط با سالمندآزاری در ادبیات فارسی شناسایی و با ساختاری آموزشی تدوین گردید در این راستا اشعار شاعران و کتاب‌های عالمان و نویسندگان ادب فارسی به کمک پایگاه اطلاعاتی درج 3 که مشتمل بر 178 جلد کتاب ادبی از 101 شاعر و نویسنده مشهور است و سایت گنجور که آثار تعداد کثیری از شاعران را در بر دارد بررسی شد و در مواردی که نسخه الکترونیک موجود نبود به مطالعه سطر به سطر کتب پرداخته شد. در نهایت با استفاده از روش کتابخانه‌ای و تحلیل محتوا مضامین مورد بررسی قرار گرفت. یافته‌ها: نتیجه پژوهش شامل تعریف سالمندآزاری انواع سالمندآزاری و علل سالمندآزاری و نحوه پیشگیری از آن بود که در متن مقاله هر کدام به تفضیل بیان شده است. نتیجه‌گیری: براساس نتایج به‌دست‌آمده از ادبیات غنی پارسی می‌توان نتیجه گرفت سالمندآزاری در ادبیات فارسی از دو جنبه مورد توجه قرار گرفته است که عبارت‌اند از: تقبیح عمل سالمندآزاری و ترویج فرهنگ تکریم و احترام به سالمند در جهت پیشگیری از سالمند آزاری.

Title: سالمندی، طول مدت اقامت و هزینه‌های بیمارستانی بیمارستان مطالعه موردی در بیمارستان میلاد تهران سال 1387 (مقاله برگرفته از پایان نامه دانشجویی)

http://salmandj.uswr.ac.ir/article-1-489-fa.html

اهداف: در بیشتر کشورهای جهان مطالعات بسیاری در ارتباط با هزینه های بستری سالمندان در بیمارستان نسبت به سایر گروه های سنی انجام شده است. هدف این مطالعه بررسی و تحقیق در رابطه با طول مدت زمان بستری بیماران سالمند و مقایسه آن با میانسالان است. مواد و روش‌ها: این پژوهش، مطالعه‌ائ از نوع توصیفی تحلیلی، مقطعی، موردی و گذشته نگر می‌باشد که بر اساس اطلاعات ثبت شده در بیمارستان میلاد از تمامی بیماران سالمند و میانسال بستری شده در سال 1387 هجری خورشیدی انجام شد. پس از تهیه جدول اطلاعات دموگرافیک شرکت کنندگان، در پژوهش آمار توصیفی مورد نیاز در خصوص هزینه تخت و طول مدت بستری در بیماران میانسال و سالمند تهیه، سپس با کمک آزمون‌های آماری (از جمله آزمون تی، آنالیز واریانس و آزمون‌های تعقیبی) نتایج به‌دست آمده تحلیل و با نتایج برخی مقالات و تحقیق های مشابه و مرتبط مقایسه شده است. یافته‌ها: در این مطالعه پس از پاکسازی اطلاعات تعداد 12989(7279) فرد سالمند با سن 8/73±60 و 5710 نفر میانسال با سن (4/17±45) که تعداد 6430 زن و 6559 مرد بودند، مورد آنالیز قرار گرفتند. بر اساس نتایج این مطالعه طول مدت زمان بستری و هزینه تخت سالمندان به‌طور معنی‌داری بیشتر از میانسالان بوده است (0/001>P). همچنین هزینه تخت در گروه سنی 60-75 سال بیشتر از سایر گروه‌های سنی سالمندان بود (0/001>P) هزینه تخت و طول مدت بستری به طور قابل ملاحظه‌ای در مردان نسبت به زنان بیشتر بوده است (0/001>P). نتیجه‌گیری: بر اساس نتایج این مطالعه سالمندی بر طول مدت بستری اثر افزاینده داشته است. در بین سالمندان بیشترین مدت اقامت به گروه 60 تا 75 ساله ها تعلق داشت. همچنین در بین سالمندان مردان نسبت به زنان مدت اقامت طولانی‌تری دارند. پیشنهاد می شود که مطالعات مداخله ای در آینده با هدف کاهش مدت بستری انجام شود، زیرا طولانی‌تر بودن زمان اقامت در بیمارستان می تواند منجر به افزایش هزینه برای سالمندان، خانواده و جامعه شده عوارض جانبی زیادی برای فرد سالمند داشته باشد.

Title: مطالعه شاخص‌های بهداشتی و اجتماعی زنان سالمند در ایران (مقاله برگرفته از پایان‌نامه دانشجویی)

http://salmandj.uswr.ac.ir/article-1-481-fa.html

اهداف: با کاهش میزان باروری و بهبود شرایط بهداشتی، اجتماعی در ایران، آمار جمعیت زنان سالمند نیز به تبعیت از کل جمعیت سالمندان افزایش‌یافته است به طوریکه در طی دو دهه نسبت جمعیت زنان سالمند به کل جمعیت زنان از 3درصد به 1/4درصد و در سرشماری اخیر در سال1385 به 7درصد افزایش یافته است و پیش‌بینی می‌شود که در طی دهه های آینده نیز این روند افزایشی ادامه یابد. آمارهای جهانی نشان می‌دهد که نسبت زنان سالمند در اکثر کشورهای جهان، پیشرفته و در حال پیشرفت، به‌طور قابل ملاحظه‌ای بیشتر از مردان سالمند است، اما بر اساس آمار سال‌های 1355-1385 مرکز آمار ایران نشان می دهد که در هر دهه به دلیل پیشرفت خدمات بهداشتی، درمانی، اجتماعی و اقتصادی تعداد زنان سالمند در ایران افزایش یافته ولی از نسبت جمعیت زنان سالمند به مردان سالمند کاسته شده است. این وضعیت در حالی اتفاق افتاده که معمولا امید به زندگی در مردان کمتر از زنان می باشد. بنابراین در این مقاله سعی شد است با مطالعه وضعیت جمعیت، سلامت، سواد، وضعیت خانوادگی، اشتغال، کیفیت زندگی، انزوای زنان سالمند در ایران دلایل متفاوت بودن نسبت جنسیتی زنان سالمند نسبت به مردان سالمند را بررسی نمائیم. مواد و روش‌ها: این مطالعه یک مطالعه توصیفی–تحلیلی و کتابخانه‌ای است که در آن از آمارهای مرکز آمار ایران که به عنوان مستندترین سرشماری کشور می‌باشد استفاده شد. علاوه بر آن database های مرتبط انگلیسی و فارسی شامل pubmed ،medline ،ISI و سایت سازمان بهداشت جهانی و پایگاه اطلاعات علمی جهاد دانشگاهی را جهت به‌دست آوردن اطلاعات دموگرافیک و اجتماعی درباره  جمعیت سالمندان ایران جستجو کردیم. همچنین در تحلیل یافته‌ها از دیدگاه‌های متخصصین سالمندشناسی، اپیدمیولوژی و جامعه‌شناسی درباره  تفاوت جنسیتی در جمعیت سالمندان ایران بهره گرفتیم. یافته‌ها: با نگاهی عمیق به وضعیت سالمندان زن از ابعاد مختلف از جمله سلامت جسمی، روحی-روانی، خانوادگی، اجتماعی و ... در می‌یابیم که علی‌رغم تلاش‌ها و پیشرفت‌هایی در وضعیت بهداشتی، اجتماعی و اقتصادی زنان ایرانی، وجود تفاوت در شاخص‌های بهداشتی–اجتماعی و جمعیتی زنان سالمند نسبت به مردان سالمند ایرانی می‌تواند به دلیل وضعیت نابرابر وضعیت بهداشتی درمانی و اجتماعی زنان نسبت به مردان باشد برخی از این تفاوت‌ها شامل: با وجود امید به زندگی بیشتر برای زنان نسبت به مردان (73 سال در برابر 68 سال)، درصد مردان سالمند از زنان سالمند بیشتر است (51/84درصد در برابر 48/16درصد) شیوع بیماری قلبی و فشار خون در زنان سالمند از مردان سالمند بیشتر است (25 و 42/6 در برابر 17 و 42درصد) شیوع افزایش چربی نوع تری‌گلیسیرید در خون در زنان سالمند از مردان بیشتر است (63/6 در برابر 42/6درصد) شیوع پوکی استخوان در زنان سالمند از مردان بیشتر است (56/3 در برابر 16/7درصد) میزان مصرف دارو در زنان سالمند از مردان بیشتر است (34/9 دربرابر 25/4درصد) میزان بیسوادی در زنان سالمند از مردان بیشتر است (80/15 در برابر 56درصد) میزان مشکلات عاطفی و روانی در زنان سالمند از مردان بیشتر است (25/3 در برابر 17/8درصد) میزان کیفیت زندگی در زنان سالمند از مردان کمتر است (31/6 در برابر 34/26درصد) میزان سپردن زنان سالمند به آسایشگاه از مردان سالمند بیشتر است (60/6 در برابر 39/4درصد) میزان تنهایی در زنان سالمند بیشتر از مردان سالمند است (12/8 در برابر 2/5درصد). نتیجه‌گیری: با توجه به نتایج فوق این‌گونه به نظر می‌رسد که سالمندان زن نه تنها به لحاظ جنسیتی در معرض آسیب قرار دارند بلکه در دوران سالمندی نیز از جهات مختلفی که در بالا ذکر شد ممکن است آنان را در معرض آسیب قرار دهد. و همین قضیه این قشر را به صورت مضاعف چه به لحاظ کمیت و چه به لحاظ کیفیت زندگی مورد تحدید قرار دهد. لذا به نظر می‌رسد در آینده انجام مطالعات ملی دقیقی در این زمینه به منظور شناخت بهتر جمعیتی سالمندی به خصوص زنان و سایر گروه‌های آسیب‌پذیر و همچنین طراحی مداخلات پیشگیری و درمان، انجام شود. با توجه به آسیب‌پذیر بودن زنان نسبت به معلولیت و عدم امنیت مالی، توجه ویژه به زنان قبل و بعد از رسیدن به سن سالمندی احساس می‌شود.
